# *GPC3* affects the prognosis of lung adenocarcinoma and lung squamous cell carcinoma

**DOI:** 10.1186/s12890-021-01549-9

**Published:** 2021-06-10

**Authors:** Jing Ning, Shenyi Jiang, Xiaoxi Li, Yang Wang, Xuhong Deng, Zhiqiang Zhang, Lijie He, Daqing Wang, Youhong Jiang

**Affiliations:** 1 Molecular Oncology Department of Cancer Research Institution, The First Hospital of China Medical University, Nanjingbei Street, Heping District, Shenyang, 110001 Liaoning Province China; 2 Department of General Medicine (VIP Ward) and Department of Tumor Supportive and Palliative Medicine, Cancer Hospital of China Medical University, Liaoning Cancer Hospital and Institute, No.44 Xiaoheyan Road, Dadong District, Shenyang, 110042 Liaoning Province China; 3 Department of General Practice, The First Hospital of China Medical University, Nanjingbei Street, Heping District, Shenyang, 110001 Liaoning Province China; 4 Central Laboratory, Cancer Hospital of China Medical University, Liaoning Cancer Hospital and Institute, No. 44 Xiaoheyan Road, Dadong District, Shenyang, 110042 Liaoning Province China; 5 The People’s Hospital of Liaoning Province, No.33 Wenyi Road, Shenhe District, Shenyang, 110016 Liaoning Province China

**Keywords:** Lung adenocarcinoma, Lung squamous cell carcinoma, Glypican 3, Differential expression analysis, Protein–protein interaction network, Risk-score model

## Abstract

**Background:**

Glypican 3 (GPC3) is a heparin sulphate proteoglycan whose expression is associated with several malignancies. However, its expression in non-small-cell lung carcinoma (NSCLC) is limited and ambiguous. This study aimed to comprehensively evaluate the expression of GPC3 in NSCLC and develop a risk-score model for predicting the prognosis of NSCLC.

**Methods:**

The gene expression profiles of lung adenocarcinoma (LUAD) and lung squamous cell carcinoma (LUSC) were downloaded from the UCSC Xena database. Using the limma package, the differentially expressed genes (DEGs) between different comparison groups were analysed and the differential expression of *GPC3* was calculated. A functional enrichment analysis was conducted for *GPC3*-associated genes using the DAVID tool. For the *GPC3*-associated genes shared by the four comparison groups, a protein–protein interaction network was built using the Cytoscape software. After conducting a survival analysis and a Cox regression analysis, the genes found to be significantly correlated with prognosis were selected to construct a risk-score model. Besides, the gene and protein levels of *GPC3* were examined by quantitative reverse transcriptase-PCR (qRT-PCR) and immunohistochemistry (IHC) in LUSC tissues and paracancer tissues.

**Results:**

The differential expression of GPC3 was significant (adjusted *P* < 0.05) in the NSCLC vs. normal, LUAD vs. normal, LUSC versus normal, and LUAD versus. LUSC comparison groups. GPC3 directly interacted with *SERPINA1, MFI2,* and *FOXM1*. Moreover, *GPC3* expression was significantly correlated with pathologic N, pathologic T, gender, and tumour stage in LUAD samples. Finally, the risk-score model (involving *MFI2*, *FOXM1*, and *GPC3*) for LUAD and that (involving *SERPINA1* and *FOXM1*) for LUSC were established separately. The qRT-PCR result showed that *GPC3* expression was much higher in the LUSC tissues than that in the normal group. The IHC results further showed that *GPC3* is highly expressed in LUSC tissues, but low in paracancer tissues.

**Conclusion:**

The three-gene risk-score model for LUAD and the two-gene risk-score model for LUSC might be valuable in improving the prognosis of these carcinomas.

## Background

Lung cancer, a malignant tumour with the fastest increasing morbidity and mortality rates, is the greatest threat to human health and life [1]. Small-cell lung carcinoma (SCLC) and non-small-cell lung carcinoma (NSCLC) are the two main pathological types of lung cancer, with NSCLC accounting for approximately 85% of lung cancers [2]. NSCLC can be categorised into several subtypes, including lung adenocarcinoma (LUAD), lung squamous cell carcinoma (LUSC), and lung large cell carcinoma (LCLC) [3]. Moreover, the occurrence of LUSC is tightly correlated with smoking, and it has been reported that the rate of exposure to smoking in LUSC patients exceeds 90% [4]. Clinically, only a small proportion of NSCLC patients is diagnosed at the early stages (stage I or II), and surgical resection is the most effective treatment for stage I, II, and IIIA NSCLC [5]. More than 60% of lung cancer patients have locally advanced or metastatic disease (stage III or IV) at the time of diagnosis and have lost the chance of radical treatment [5]. In patients who have undergone surgical treatment, there is a high risk of recurrence despite the possibility of complete remission. Therefore, it is important to understand the pathogenesis of NSCLC to improve treatment outcomes.

The past decades have witnessed rapid development in the pathology of lung cancer, and numerous dysregulated genes involved in NSCLC have been identified. A previous study demonstrated that astrocyte-elevated gene-1 (*AEG-1*) acts in the formation and deterioration of NSCLC by regulating matrix metalloproteinase-9 (*MMP9*), resulting in an unfavourable clinical outcome [6]. Glyceraldehyde-3-phosphate dehydrogenase (*GAPDH*) overexpression indicates a poor prognosis in early-stage NSCLC, and the assessment of glucose metabolism has certain prognostic value in this tumour [7]. Hypoxia-inducible factor-2α (*HIF-2α*) expression is correlated with lymph-node metastasis, tumour size, tumour histology, and tumour stage, making it a potential candidate target for predicting the progression and clinical outcome of LUAD [8, 9]. Decreased N-MYC downstream-regulated gene 2 (*NDRG2*) is important for the tumorigenesis of lung cancer and may be considered a valuable prognostic marker in lung cancer [10]. Increased Notch homolog 2 (*Notch2* expression in LUAD patients can induce a high tumour recurrence rate, and high expression of Notch1 and Notch3 is related to adverse prognosis in LUAD [11]. Claudin-3 (*CLDN3*) in LUSC tissues is related to tumour progression and represses epithelial–mesenchymal transition (EMT) via activation of the Wnt pathway; therefore, *CLDN3* may be a candidate biomarker for the prognosis and treatment of LUSC [12]. However, more genes affecting the prognosis of NSCLC still need to be explored.

Glypican 3 (*GPC3*) is a membrane-bound heparin sulphate proteoglycan located on chromosome Xq26 [13]. It is highly expressed during foetal life, but its levels decrease after birth [14]. The expression patterns of *GPC3* in different cancer types have been reported to be different, and its role is controversial. *GPC3* is overexpressed in hepatocellular carcinoma (HCC), embryonal tumours, melanoma, hepatoblastoma, and testicular germ-cell tumours, and it acts as a tumour oncogene [15–20]. However, mutations or loss of expression have been reported in Simpson-Golabi-Behmel syndrome [21], ovarian carcinoma, breast cancer, and mesothelioma [22–25], suggesting that *GPC3* functions as a tumour-suppressor gene. Currently, reports of GPC3 in lung cancer are limited and ambiguous. Kim et al. reported that *GPC3* expression was decreased in LUAD compared with that in paired normal tissues [26]. In a study by Sarit et al., GPC3 was found to be overexpressed in LUSC (positive rate of 55%) but not in LUAD (positive rate of 8%), which might be induced by smoking [27].

In this study, the gene expression profiles of NSCLC were obtained. Differential expression and enrichment analyses for different comparison groups were then carried out. After the genes correlated with *GPC3* were screened out, a protein–protein interaction (PPI) network analysis, survival analysis, and Cox regression analysis were conducted separately. The present results might help to elucidate the *GPC3*-correlated prognostic mechanisms of LUAD and LUSC.

## Methods

### Data source

From the University of California Santa Cruz (UCSC) Xena database (https://xenabrowser.net/datapages/), the gene expression profiles (standardised expression values of log_2_[fragments per kilobase of transcript per million reads (FPKM) + 1]) of LUAD (including 526 tumour samples and 59 normal samples) and LUSC (including 501 tumour samples and 49 normal samples) were downloaded. Meanwhile, the clinical phenotypes (including smoking and sex) and prognostic information (including survival status and survival time) of these samples were extracted. These samples all contain clinical phenotypes and prognostic information.

### Differential expression analysis and enrichment analysis

The samples were divided into seven comparison groups: NSCLC versus normal, LUAD versus normal, LUSC versus normal, LUAD versus LUSC, male versus female, smoker versus non-smoker, LUSC smoker versus LUSC non-smoker. A differential expression analysis was conducted using the R package limma [28] (version 3.10.3, http://www.bioconductor.org/packages/2.9/bioc/html/limma.html), and the *P *values of the genes were adjusted using the Benjamini & Hochberg method [29]. To screen the differentially expressed genes (DEGs), |logfold change (FC)|> 1 and adjusted *P* < 0.05 were defined.

For the DEGs of each comparison group, Gene Ontology (GO) and Kyoto Encyclopaedia of Genes and Genomes (KEGG) [30, 31] enrichment analyses were performed separately using the DAVID online tool [32] (version 6.7, https://david-d.ncifcrf.gov/). A false discovery rate (FDR) < 0.05 was the threshold for selecting significantly enriched results.

### Construction of PPI network

For each comparison group, the genes involved in the significant GO/KEGG terms correlated with *GPC3* were screened. Then, the intersections of these genes in different comparison groups were selected by drawing a Venn diagram [33], and the common genes were considered candidate genes. Using the STRING database [34] (http://www.string-db.org) and Cytoscape software [35] (https://cytoscape.org/), a PPI network was constructed to identify the hub genes and the genes directly correlated with *GPC3*.

### Correlation of GPC3 with clinical phenotypes

There were 585 LUAD and 550 LUSC samples. In addition to *GPC3* expression, phenotypes such as age, location, years smoked, pathologic M, pathologic N, pathologic T, radiation therapy, sex, and tumour stage were also investigated. The baseline data of LUAD and LUSC samples are listed in Tables 1 and 2, respectively. *GPC3* expression is the standardised expression value of log_2_(FPKM + 1). The average value of the non-empty samples was calculated for the expression of two characteristics—years smoked and *GPC3* expression. The non-empty samples were then compared with the average value and divided into two groups: high and low. Lung cancer was most common between the ages of 45 and 65 years; therefore, the samples were divided into two groups based on age (≥ 65 or < 65 years).

**Table 1:** Baseline data of lung adenocarcinoma (LUAD) samples

Characteristics	Number	Percent (%)
*Age*		
≥ 65	310	53.0
< 65	256	43.8
NA	19	3.2
*Location*		
Central lung	67	11.5
Peripheral lung	130	22.2
NA	388	66.3
*Years_smoked*		
≥ 41	160	27.4
< 41	234	40.0
NA	191	32.6
*pathologic_M*		
M0	394	67.4
M1	20	3.4
M1a	2	0.3
M1b	5	0.9
MX	158	27.0
NA	6	1.0
*pathologic_N*		
N0	371	63.4
N1	107	18.3
N2	87	14.9
N3	2	0.3
NX	17	2.9
NA	1	0.2
*pathologic_T*		
T1	82	14.0
T1a	49	8.4
T1b	60	10.3
T2	203	34.7
T2a	89	15.2
T2b	29	5.0
T3	50	8.5
T4	20	3.4
TX	3	0.5
*radiation_therapy*		
Yes	69	11.8
No	428	73.2
NA	88	15.0
*Gender*		
Female	316	54.0
Male	269	46.0
*tumor_stage*		
I	316	54.0
II	135	23.1
III	97	16.6
IV	28	4.8
NA	9	1.5
*GPC3 expression*		
≥ 3.19	270	46.2
< 3.19	315	53.8

NA represents the sample with an empty record

**Table 2:** Baseline data of lung squamous cell carcinoma (LUSC) samples

Characteristics	Number	Percent (%)
*Age*		
≥ 65	355	54.5
< 65	186	33.8
NA	9	1.6
*Location*		
Central Lung	157	28.5
Peripheral Lung	101	18.4
NA	292	53.1
*years_smoked*		
≥ 53	177	32.2
< 53	287	52.2
NA	86	15.6
*pathologic_M*		
M0	443	80.5
M1	6	1.1
M1a	1	0.2
M1b	1	0.2
MX	94	17.1
NA	5	0.9
*pathologic_N*		
N0	352	64.0
N1	143	26.0
N2	43	7.8
N3	5	0.9
NX	7	1.3
*pathologic_T*		
T1	53	9.6
T1a	26	4.7
T1b	44	8.0
T2	187	34.0
T2a	100	18.2
T2b	40	7.3
T3	76	13.8
T4	24	4.4
*radiation_therapy*		
Yes	51	9.3
No	386	70.2
NA	113	20.5
*Gender*		
Female	144	26.2
Male	406	73.8
*tumor_stage*		
I	270	49.1
II	179	32.5
III	89	16.2
IV	8	1.5
NA	4	0.7
*GPC3 Expression*		
≥ 4.48	289	52.5
< 4.48	261	47.5

NA represents the sample with an empty record

For each clinical phenotype, *GPC3* expression was correlated with the clinical phenotype subgroups of the samples. The Wilcoxon rank sum test [36] was conducted for the phenotypes of the two groups, and the Kruskal–Wallis rank sum test [37] was performed for the phenotypes of multiple groups. A *P *value < 0.05 was set as the threshold.

### Survival analysis

Based on the extracted prognostic information of the samples, the overall survival (OS) and OS status of the corresponding patients were determined. *GPC3* and the genes directly correlated with *GPC3* were considered as candidate features, and the patients were classified into high-expression and low-expression groups based on the median expression value of *GPC3*. The median expression value greater than *GPC3* was high-expression, and the median expression value less than or equal to *GPC3* was low-expression. Combined with the prognostic information of the samples, Kaplan–Meier (KM) survival analysis [38] was carried out. The log-rank test [39] was used to calculate *P *values. A *P *value < 0.05 indicated a significant correlation.

### Univariate and multivariate Cox regression analyses

Based on the expression levels of *GPC3* and the genes directly correlated with *GPC3,* along with the prognostic information of the samples, univariate Cox regression analysis [40] was performed using the coxph() function in R [41]. The regression coefficient and *P *value of each clinical factor, survival time, and state were calculated. Subsequently, a multivariate Cox regression analysis [42] was conducted for the clinical factors with *P* < 0.05, to obtain the final risk-score model. The samples were divided into high-risk and low-risk groups based on their risk scores, and a KM survival analysis [43] was performed. Furthermore, the 1-year, 3-year, and 5-year survival rates of the samples were predicted based on their risk scores; receiver operating characteristic (ROC) curves [44] were drawn, and the corresponding area under the ROC curve (AUC) values were calculated.

### cBioPortal analysis

Genome data from the Cancer Genome Atlas (TCGA) lung cancer dataset using cBioportal (https://www.cbioportal.org/) have been retrieved in order to identify mutations and copy number alterations (CNAs) of *GPC3* [45]. The location and frequency of *GPC3* and *GPC3*-related gene alterations (amplifications, deep deletions and missene mutations) and copy number variance data were evaluated.

### Quantitative reverse transcriptase-PCR (qRT-PCR)

Total RNA was extracted from LUSC and paracancer tissues using the TRIzol Reagent (Invitrogen, USA). Then, the cDNA was reverse-transcribed using a PrimeScriptTM RT kit with gDNA Eraser (TaKaRa, China). According to the manufacturer’s instructions, qRT-PCR was performed using TB Green® Premix Ex Taq™ II (Takara, Japan) on a CFX96 Real-Time PCR Detection System. GAPDH was used as an internal reference gene. The reaction mixture for qRT-PCR was prepared as follows: 8.5 μL of sterile purified water, 12.5 μL of TB Green Premix Ex Taq II (Tli RNaseH Plus) (2X), 1 μL of PCR forward primer (10 μM), 1 μL of qRT-PCR reverse primer (10 μM), and 2 μL cDNA template (< 100 ng) were mixed. The reaction conditions for qRT-PCR were as follows: initial denaturation at 95 °C for 30 s, followed by 40 cycles of 95 °C for 5 s and 60 °C for 30 s for denaturation and annealing/elongation, respectively. The 2^−ΔΔCT^ method was used to measure relative expression.

### Immunohistochemistry (IHC) validation

Cancer tissue specimens and paraffin sections of adjacent tissues were collected from 10 patients undergoing pulmonary malignant tumour surgery at the Liaoning Cancer Hospital and Institute between June 2018 and June 2020. The study was approved by the Ethics Committee of the Liaoning Cancer Hospital and Institute. The paraffin specimens of LUSC patients obtained after surgery were cut into two pieces for IHC staining. The sections were deparaffinized, and antigen retrieval was performed with citrate buffer (pH 6.0) under high temperature and pressure, followed by natural cooling to room temperature and dilution with PBS. After incubation in 3% BSA for 1 h, the cells were incubated with an Anti-GPC3 antibody overnight at 4 °C. Subsequently, the sections were incubated with secondary antibodies (Zsbio, China) and stained with diaminobenzidine (DAB) and haematoxylin.

## Results

### Differential expression and enrichment analyses

**Fig. 1:**
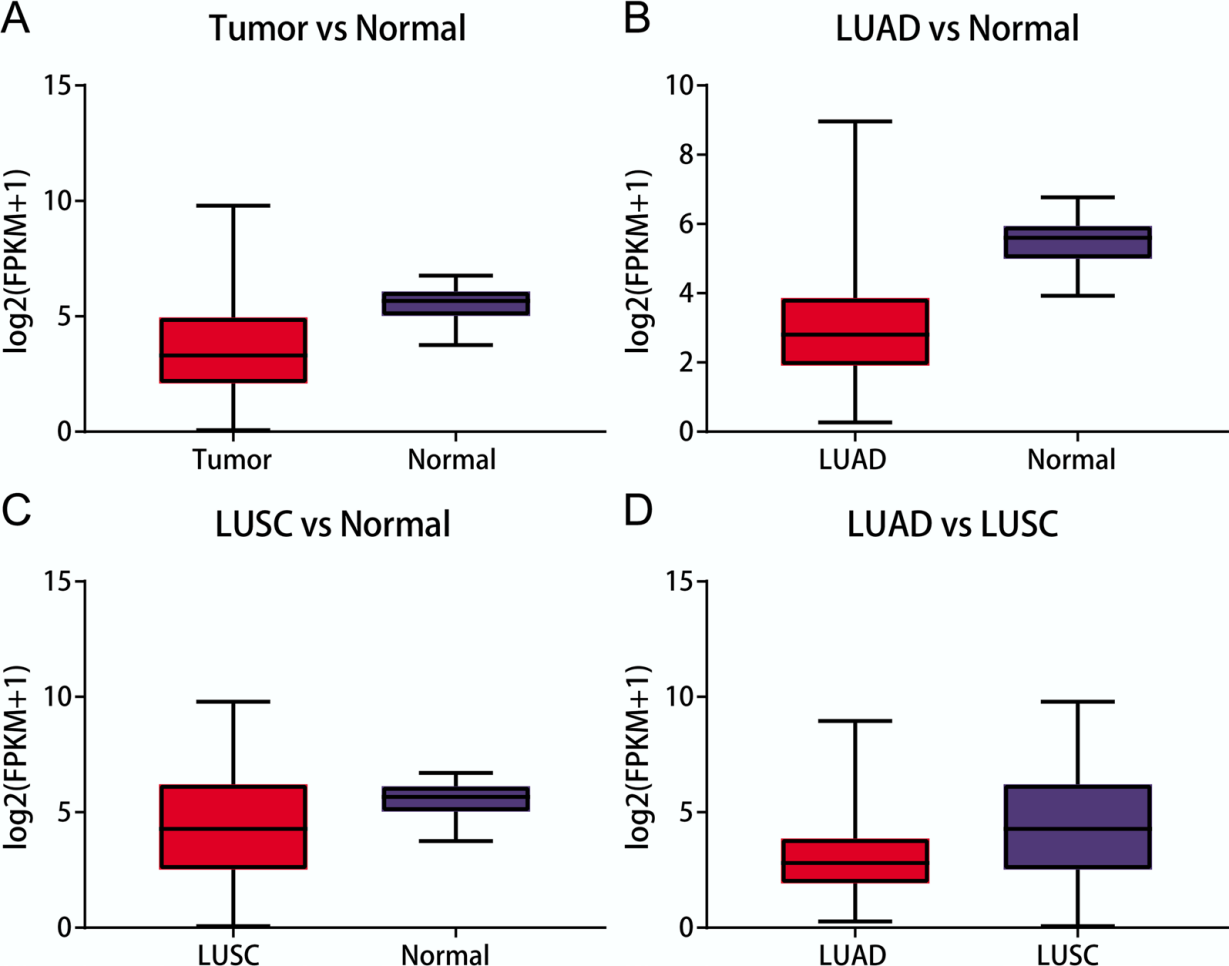
*GPC3* is differentially expressed in all of the four comparison groups (*P* < 0.05 and |logFC|> 1). **a** Differential expression of *GPC3* in the lung cancer vs. normal comparison group; **b** Differential expression of *GPC3* in the lung adenocarcinoma (LUAD) vs. normal comparison group; **c** Differential expression of *GPC3* in the lung squamous cell carcinoma (LUSC) versus normal comparison group; **d** Differential expression of *GPC3* in the LUAD versus LUSC comparison group

In total, 2478 DEGs (1324 up-regulated and 1154 down-regulated genes) in the NSCLC vs. normal comparison group, 1998 DEGs (904 up-regulated and 1094 down-regulated genes) in the LUAD vs. normal comparison group, 3425 DEGs (1670 up-regulated and 1755 down-regulated genes) in the LUSC vs. normal comparison group, 1072 DEGs (488 up-regulated and 584 down-regulated genes) in the LUAD vs. LUSC comparison group, 63 DEGs (43 up-regulated and 20 down-regulated genes) in the male vs. female comparison group, and 17 DEGs (14 up-regulated and 3 down-regulated genes) in the smoker vs non-smoker comparison group were identified. There was no difference between the LUSC smoker and LUSC non-smoker groups. Among these seven comparison groups, the difference in *GPC3* expression only reached a significant level (adjusted *P* < 0.05) in four comparison groups (Fig. 1). Therefore, the DEGs in these four groups were selected for further analysis.

There were 134 GO/KEGG terms (such as response to wounding, immune response, and cell adhesion, involving 1513 genes) enriched for the DEGs in the NSCLC vs. normal, 105 terms (such as response to wounding, cell adhesion, and biological adhesion, involving 1113 genes) in the LUAD vs. normal, 133 terms (such as response to wounding, mitosis, and nuclear division, involving 1701 genes) in the LUSC vs. normal, and 38 terms (such as ectoderm development, epidermis development, and epithelium development, involving 563 genes) in the LUAD versus LUSC comparison groups. Among these, 19 terms (such as regulation of cell proliferation, tube development, and epithelium development, involving 1015 genes) in the NSCLC versus normal comparison group, 17 terms (such as regulation of cell proliferation, tube development, and branching morphogenesis of a tube, involving 803 genes) in the LUAD versus normal comparison group, 18 terms (such as regulation of cell proliferation, tube development, and epithelium development, involving 1128 genes;) in the LUSC versus normal comparison group, and 12 terms (such as epithelium development, regulation of cell proliferation, and tube morphogenesis, involving 473 genes) in the LUAD versus LUSC comparison group were correlated with *GPC3*.

### Construction of PPI network

**Fig. 2:**
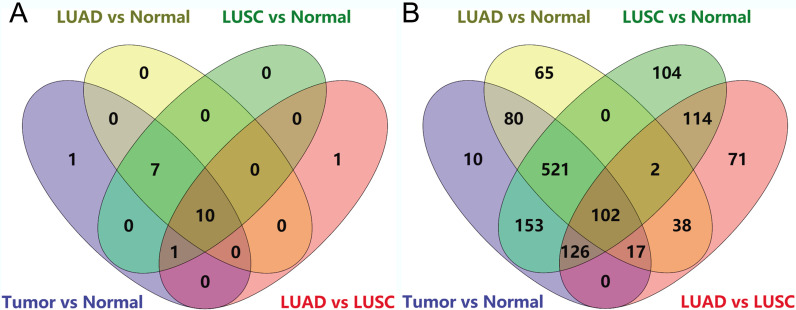
Venn diagrams showing *GPC3*-associated enrichment terms and genes shared by the four comparison groups. **a** The enrichment terms shared by the four comparison groups; **b** The genes shared by the four comparison groups

**Fig. 3:**
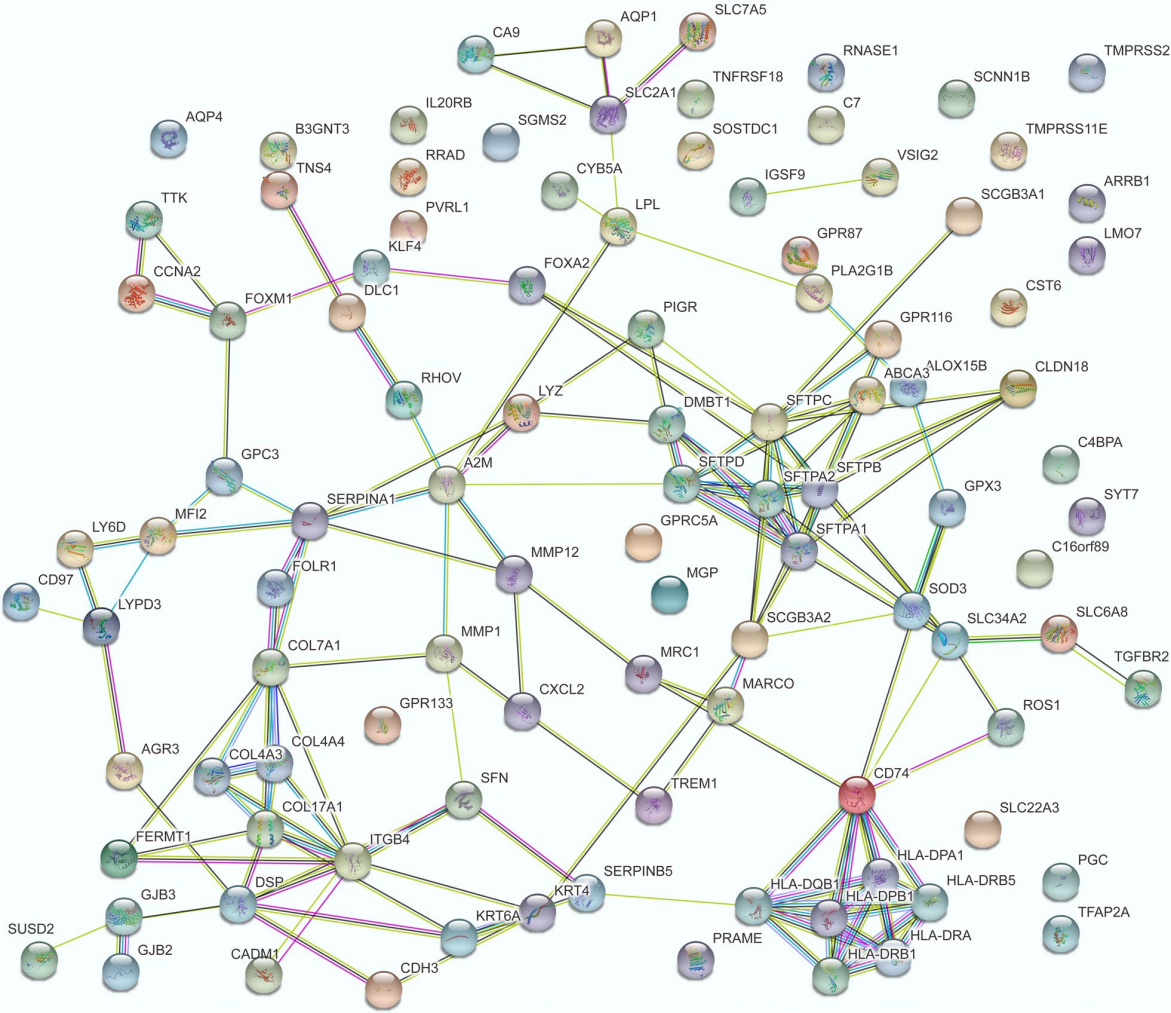
Protein–protein interaction network for the 102 common genes

Venn diagrams showed that the 10 GO/KEGG terms (such as regulation of cell proliferation, plasma membrane, and extracellular space) (Fig. 2a) and 102 genes (Fig. 2b) correlated with *GPC3* were shared by the four comparison groups. For the 102 common genes (including *GPC3*), PPI pairs were predicted, and the PPI network (including 148 edges) was visualised (Fig. 3). In the PPI network, GPC3 directly interacted with serpin family A member 1 (SERPINA1), melanin transferrin (MFI2), and forkhead box M1 (FOXM1).

### Correlation of GPC3 with clinical phenotypes

**Fig. 4:**
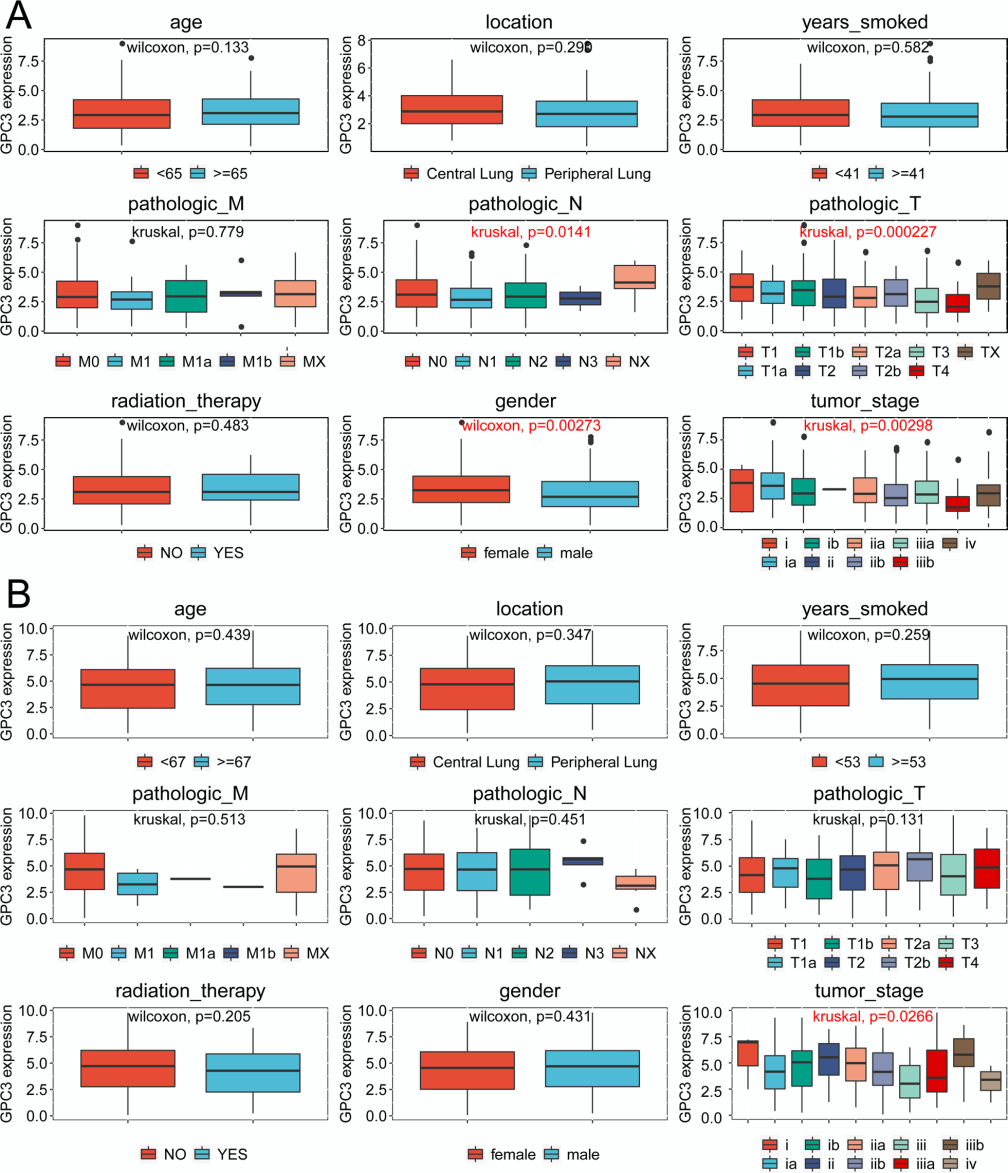
Correlation of *GPC3* expression with clinical phenotypes (*P* < 0.05). **a** Correlation of *GPC3* expression in the LUAD samples with clinical phenotypes; **b** Correlation of *GPC3* expression in the LUSC samples with clinical phenotypes

The results showed that *GPC3* expression in LUAD samples was significantly correlated with pathologic N (*P* = 0.01405), pathologic T (*P* = 0.000227), sex (*P* = 0.002734), and tumour stage (*P* = 0.04348), but was not correlated with age, location, years smoked, pathologic M, or radiation therapy (*P* > 0.05) (Table 3, Fig. 4a). Nevertheless, *GPC3* expression in LUSC samples was not significantly correlated with age, location, years smoked, pathologic M, pathologic N, pathologic T, radiation therapy, sex, or tumour stage (*P* > 0.05) (Table 4, Fig. 4b).

**Table 3:** Correlation of GPC3 expression with the clinical phenotypes of lung adenocarcinoma (LUAD) samples

Characteristics	Variable	Number of patients	GPC3 expression				Test	*P* value
			High	Percent (%)	Low	Percent (%)		
Age	≥ 65	310	146	55.9	164	53.8	Wilcoxon	0.1332
	< 65	256	115	44.1	141	46.2		
Location	Central Lung	67	29	39.2	38	30.9	Wilcoxon	0.2896
	Peripheral Lung	130	45	60.8	85	69.1		
years_smoked	≥ 41	160	105	62.5	129	57.1	Wilcoxon	0.5824
	< 41	234	63	37.5	97	42.9		
pathologic_M	M0	394	1	0.4	1	0.3	Kruskal	0.7793
	M1	20	3	1.1	2	0.6		
	M1a	2	6	2.3	14	4.4		
	M1b	5	176	66.7	218	69.2		
	MX	158	78	29.5	80	25.4		
pathologic_N	N0	371	14	5.2	3	1	Kruskal	0.0141
	N1	107	177	65.8	194	61.6		
	N2	87	38	14.1	69	21.9		
	N3	2	39	14.5	48	15.2		
	NX	17	1	0.4	1	0.3		
pathologic_T	T1	82	24	8.9	25	7.9	Kruskal	0.0002
	T1a	49	34	12.6	26	8.3		
	T1b	60	34	12.6	55	17.5		
	T2	203	4	1.5	16	5.1		
	T2a	89	14	5.2	15	4.8		
	T2b	29	91	33.7	112	35.6		
	T3	50	16	5.9	34	10.8		
	T4	20	51	18.9	31	9.8		
	TX	3	2	0.7	1	0.3		
Radiation therapy	Yes	69	33	13.9	36	13.9	Wilcoxon	0.4830
	No	428	205	86.1	223	86.1		
Gender	Female	316	110	40.7	159	50.5	Wilcoxon	0.0027
	Male	269	160	59.3	156	49.5		
Tumor stage	I	316	163	61.5	153	49.2	Kruskal	0.0435
	II	135	11	4.2	17	5.5		
	III	97	50	18.9	85	27.3		
	IV	28	41	15.5	56	18

**Table 4:** Correlation of GPC3 expression with the clinical phenotypes of lung squamous cell carcinoma (LUSC) samples

Characteristics	Variable	Number of patients	GPC3 expression				Test	*P* value
			High	Percent (%)	Low	Percent (%)		
Age	≥ 65	355	183	64.9	172	66.4	Wilcoxon	0.9002
	< 65	186	99	35.1	87	33.6		
Location	Central Lung	157	83	57.2	74	65.5	Wilcoxon	0.3471
	Peripheral Lung	101	62	42.8	39	34.5		
years_smoked	≥ 53	177	146	59.1	141	65	Wilcoxon	0.2589
	< 53	287	101	40.9	76	35		
pathologic_M	M0	443	0	0	1	0.4	Kruskal	0.5125
	M1	6	0	0	1	0.4		
	M1a	1	1	0.3	5	1.9		
	M1b	1	235	82.2	208	80.3		
	MX	94	50	17.5	44	17		
pathologic_N	N0	352	1	0.3	6	2.3	Kruskal	0.4514
	N1	143	189	65.4	163	62.5		
	N2	43	73	25.3	70	26.8		
	N3	5	22	7.6	21	8		
	NX	7	4	1.4	1	0.4		
pathologic_T	T1	53	15	5.2	11	4.2	Kruskal	0.1308
	T1a	26	20	6.9	24	9.2		
	T1b	44	59	20.4	41	15.7		
	T2	187	14	4.8	10	3.8		
	T2a	100	26	9	14	5.4		
	T2b	40	96	33.2	91	34.9		
	T3	76	34	11.8	42	16.1		
	T4	24	25	8.7	28	10.7		
Radiation therapy	Yes	51	24	10.4	27	13.1	Wilcoxon	0.2045
	No	386	207	89.6	179	86.9		
Gender	Female	144	216	74.7	190	72.8	Wilcoxon	0.4309
	Male	406	73	25.3	71	27.2		
Tumor stage	I	270	144	50.2	126	48.6	Kruskal	0.3464
	II	179	1	0.3	7	2.7		
	III	89	94	32.8	85	32.8		
	IV	8	48	16.7	41	15.8		

### Survival analysis

**Fig. 5:**
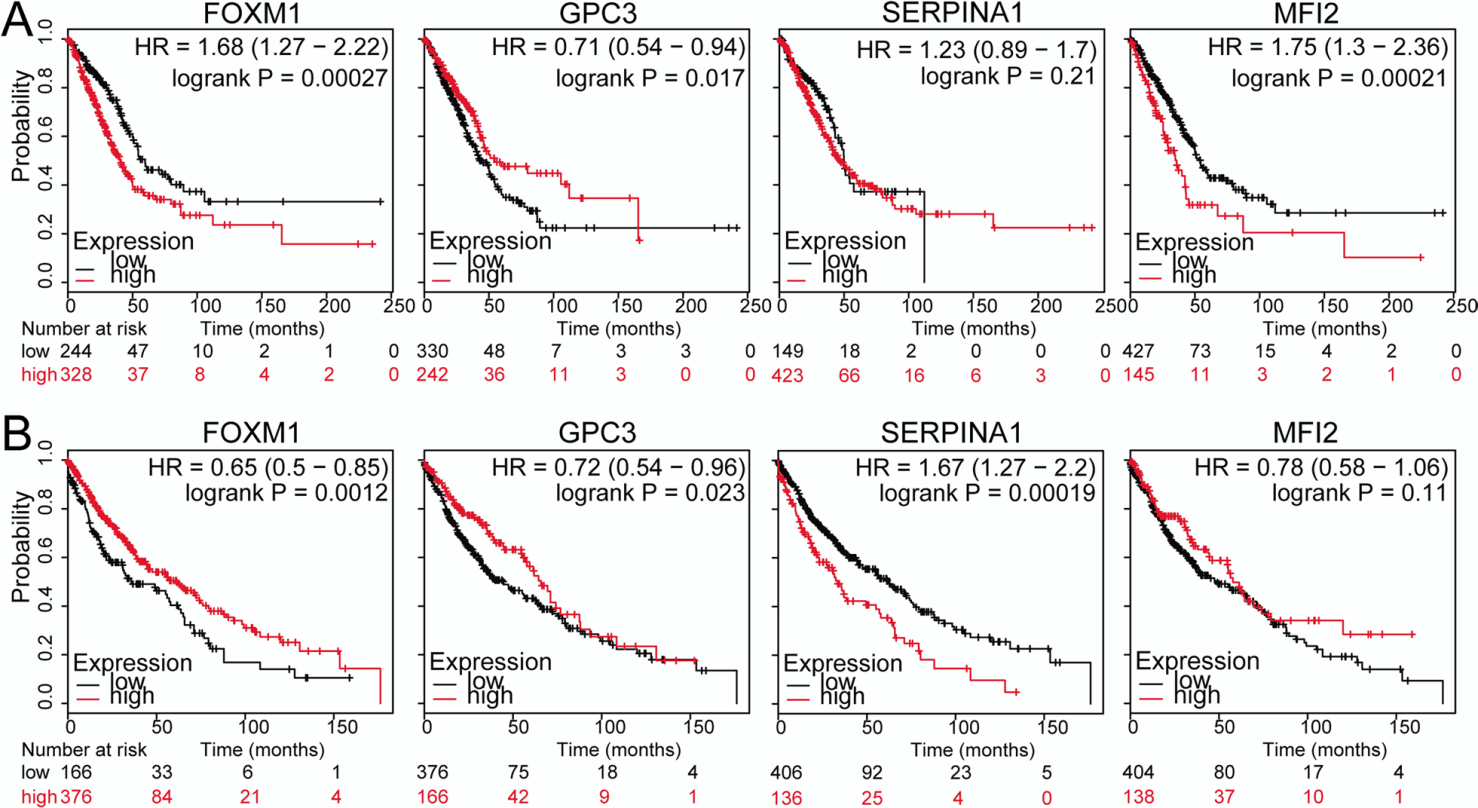
Kaplan–Meier (KM) survival curves of *FOXM1*, *GPC3*, *SERPINA1*, and *MFI2* (*P* < 0.05). **a** Survival curves for the four genes in the LUAD samples; **b** Survival curves for the four genes in the LUSC samples. Black and red curves represent low and high expression, respectively

For *GPC3* and the genes (including *SERPINA1*, *MFI2*, and *FOXM1*) directly interacting with it, gene expression levels were correlated with the prognostic information of the samples to perform KM survival analysis separately for LUAD and LUSC. The results showed that *GPC3*, *MFI2*, and *FOXM1* were significantly correlated with the prognosis of LUAD patients (Fig. 5a), and *GPC3*, *SERPINA1*, and *FOXM1* were significantly correlated with the prognosis of LUSC patients (Fig. 5b).

### Univariate and multivariate Cox regression analyses

The results of univariate Cox regression analysis showed that *MFI2*, *FOXM1*, and *GPC3* had a significant influence on the survival time of LUAD patients (*P* < 0.05, Table 5). A multivariate Cox regression analysis further suggested that the combination of *MFI2*, *FOXM1*, and *GPC3* could significantly affect the prognosis (*P-*values of the likelihood ratio test, Wald test, and score (log rank) test were 2.908e−05, 1.105e−05, and 8.474e−06, respectively). Finally, the following risk-score model was established: Risk score = 0.26187*MFI2 + 0.07721*FOXM1—0.01199*GPC3.

**Table 5:** Results of the univariate Cox regression analysis of lung adenocarcinoma (LUAD) samples

Gene	Coef	Exp (coef)	SE (coef)	z	*P* value
*MFI2*	0.3160	1.3716	0.0637	4.96	7.20E−07
*SERPINA1*	0.0027	1.0027	0.0399	0.07	9.50E−01
*FOXM1*	0.1871	1.2058	0.0538	3.48	5.00E−04
*GPC3*	− 0.0996	0.9052	0.0459	− 2.17	3.00E−02

**Fig. 6:**
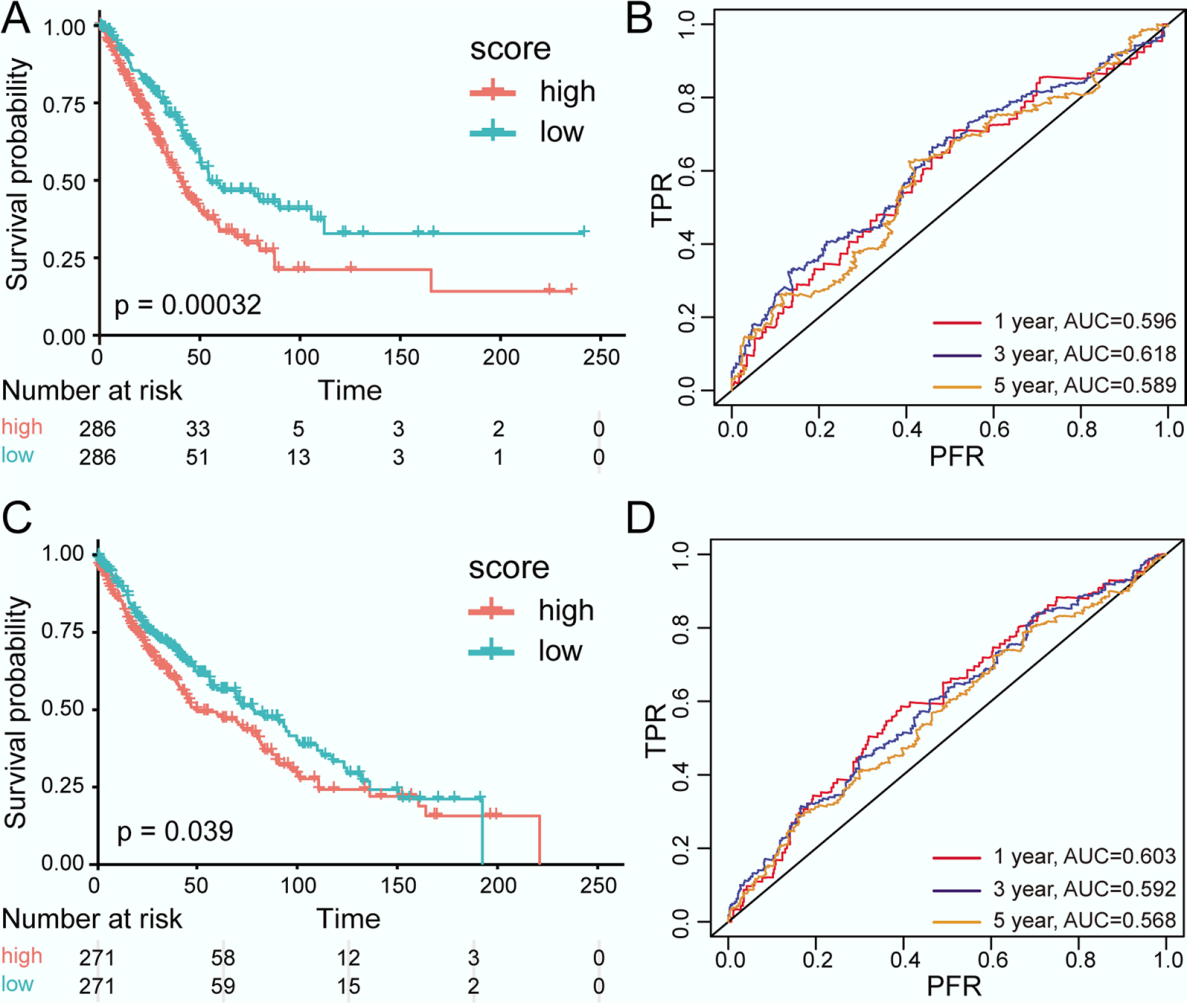
Kaplan–Meier (KM) survival curves and receiver operating characteristic (ROC) curves (*P* < 0.05). **a** KM curves for the LUAD samples; **b** ROC curves for the LUAD samples; **c** KM curves for the LUSC samples; **d** ROC curves for the LUSC samples. In the KM curves, red and green represent high and low risk, respectively. In the ROC curves, red, blue, and orange represent 1-year, 3-year, and 5-year survival situations, respectively

Combined with the risk score model, the samples were classified into high-risk and low-risk groups. A KM survival analysis indicated that the survival time of the low-risk group was significantly higher than that of the high-risk group (*P* = 0.00032, Fig. 6a). The AUC values of the 1-year, 3-year, and 5-year survival situations predicted by the risk-score model were stabilised at about 0.6 (Fig. 6b).

Meanwhile, a univariate Cox regression analysis showed that *SERPINA1* and *FOXM1* had significant effects on the survival time of LUSC patients (*P* < 0.05, Table 6). Moreover, *SERPINA1* and *FOXM1* were included in the multivariate Cox regression analysis, and the *P *values of the likelihood ratio test, Wald test, and score (log rank) test were 0.002019, 0.002015, and 0.001904, respectively. In addition, the following risk-score model was constructed: Risk score = 0.12417*SERPINA1 + 0.00518*FOXM1.

**Table 6:** Results of the univariate Cox regression analysis of lung squamous cell carcinoma (LUSC) samples

Gene	Coef	Exp (coef)	SE (coef)	z	*P* value
*MFI2*	−0.0660	0.9361	0.0505	−1.31	0.1900
*SERPINA1*	0.1219	1.1296	0.0346	3.53	0.0004
*FOXM1*	−0.0971	0.9075	0.0474	−2.05	0.0410
*GPC3*	−0.0165	0.9837	0.0301	−0.55	0.5900

The LUSC samples were divided into high-risk and low-risk groups based on the risk-score model, and a KM survival analysis showed that the survival time of the low-risk group was significantly higher than that of the high-risk group (*P* = 0.039, Fig. 6c). Similarly, all AUC values of the 1-year, 3-year, and 5-year survival situations predicted by the risk-score model were approximately 0.6 (Fig. 6d).

### Genomic alterations of GPC3 and GPC3-related gene

**Fig. 7:**
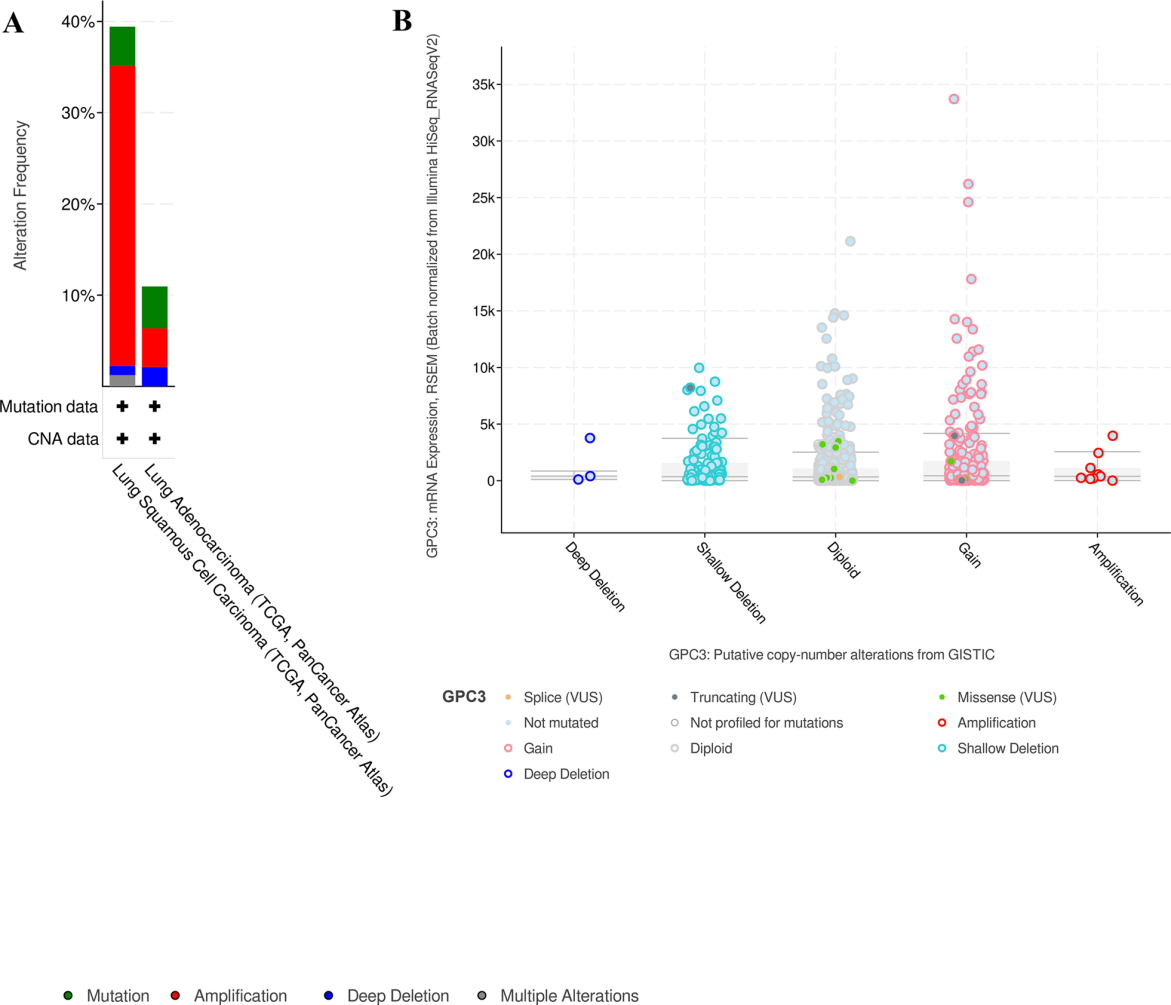
*GPC3* and *GPC3*-related genes mutations and CNAs in cBioportal for Cancer Genomics database. **a** The alteration frequency of *GPC3* and GPC3-related genes (*SERPINA1*, *MFI2* and *FOXM1*) was explored in LUSC and LUAD in 2 studies. **b** Relative *GPC3* expression mRNA level as a function of relative copy number alteration were plotted in one specific LUSC and LUAD database (TCGA, PanCancer Atlas)

TCGA datasets of all lung cancer samples were chosen to examine mutations and CNAs in the *GPC3* and *GPC3*-related genes. For this analysis, a total of 1053 cases in 2 studies were included (Fig. 7). In LUSC, 4.31% (21 cases) were mutation, 32.85% (160 cases) were amplification, 1.03% (5 cases) were deep deletion, and 1.23% (6 cases) were multiple alteration among 39.43% of 487 cases. However, in LUAD, the overall alteration frequency of *GPC3* and *GPC3*-related genes was 10.95% of 560 cases, the mutation frequency was 4.59% (26 cases), the amplification frequency was 4.06% (23 cases), and the deep deletion frequency was 2.3%. (13 cases). Moreover, mutation analysis revealed the individual alteration frequencies of *GPC3* (13 missense and 4 truncating mutations), *SERPINA1* (7 missense and 2 truncating mutations), *MFI2* (7 missense and 6 truncating mutations), and *FOXM1* (15 missense and 1 truncating mutations).

### GPC3 expression analysis with qRT-PCR

**Fig. 8:**
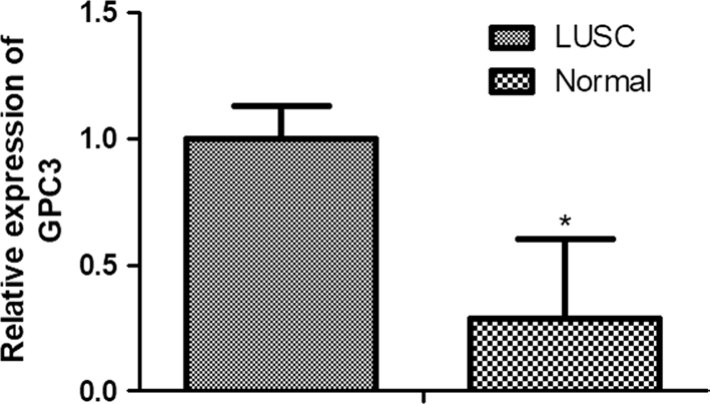
Relative mRNA expression by quantitative real-time PCR of GPC3 in LUSC compared with control group, *P < 0.05

The qRT-PCR was used to determine the expression of *GPC3* in LUSC. *GPC3* expression was much higher in the LUSC tissues than that in the control group (*P* < 0.001, Fig. 8). This is consistent with the previous sequencing results.

### IHC analysis of GPC3 protein expression

**Fig. 9:**
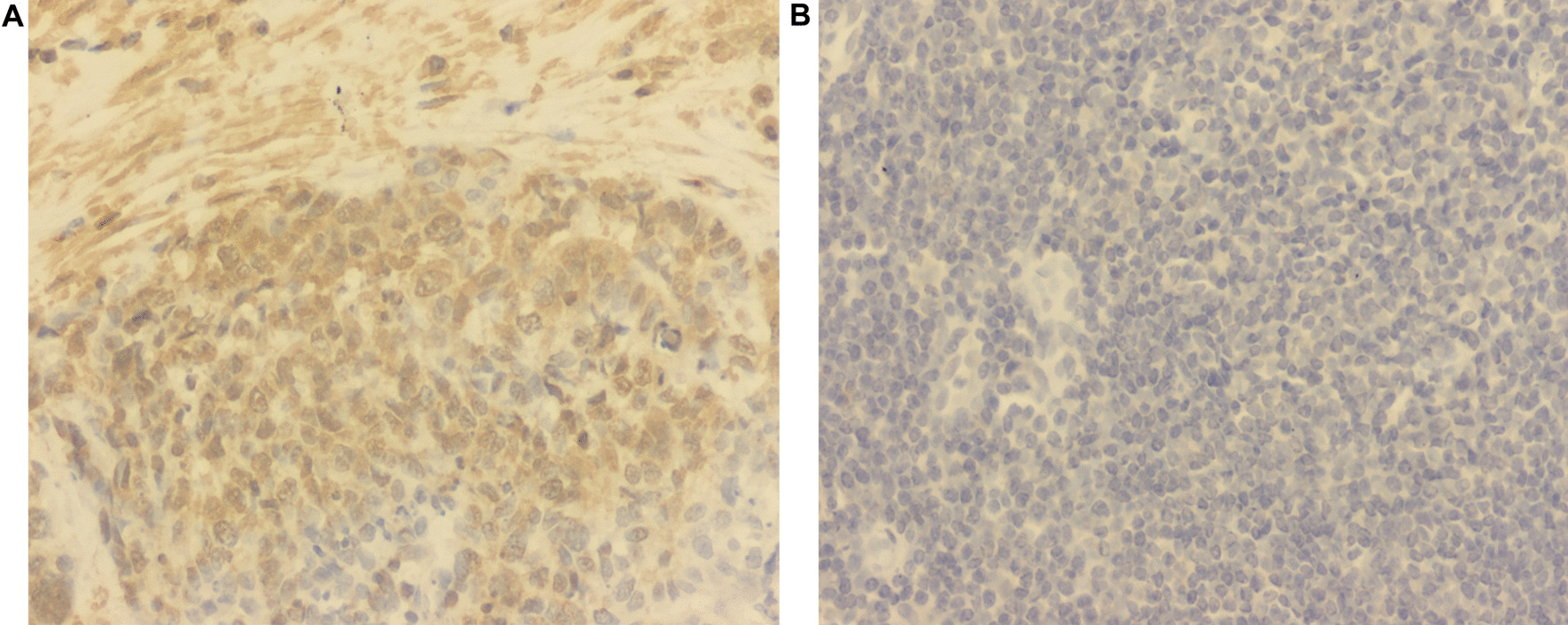
Immunohistochemical evaluation of GPC3 expression in representative luteal phase specimens of **a** LUSC and **b** paracancer tissues

IHC analysis revealed positive staining of *GPC3* protein in seven of the ten (70%) paraffin-embedded LUSC tissues, while negative staining was observed in the remaining cases (three of ten, 30%) (Fig. 9). This suggests that the high expression of *GPC3* may be related to the occurrence of LUSC.

## Discussion

*GPC3* is a heparin sulphate proteoglycan whose expression is associated with several malignancies. However, its expression in lung cancer is limited and remains ambiguous. This study found that *GPC3* is significantly downregulated in NSCLC tissues compared with that in paracancer tissues, and its expression in LUAD is significantly lower than that in LUSC. In LUAD samples, *GPC3* expression was significantly correlated with pathologic N, pathologic T, gender, and tumour stage. However, *GPC3* expression was not significantly associated with these clinical phenotypes in LUSC.

*GPC3* overexpression in liver cancer has been frequently reported without debate; however, its expression pattern in NSCLC remains debatable. Kim et al. reported that *GPC3* expression was decreased in LUAD compared with that in paired normal tissues [26]. In a study by Sarit et al., *GPC3* was found to be overexpressed in LUSC (positive rate of 55%) but not in LUAD (positive rate of 8%), which might be induced by smoking [27]. The present study found that the expression of *GPC3* in NSCLC is lower than that in paracancer tissues, which is in line with a study by Kim et al. [26] and in contradiction with a study by Sarit et al. In addition, the present study found that *GPC3* was not differentially expressed in the smoker vs. non-smoker group or the LUSC smoker vs. LUSC non-smoker group. This is also different from the study by Sarit et al. It was hypothesised that this is due to the use of different methods to detect *GPC3* expression and the sample size. Sarit et al. used immunohistochemistry on tissue microarrays to evaluate the expression of *GPC3* in 97 patients. However, the present study was based on high-sequencing data of more than 500 samples in TCGA database.

The molecular mechanism of action of *GPC3* in cancer remains unclear. In this study, a PPI network was developed for *GPC3*-associated genes. In this network, *GPC3* exhibited significant correlation with *SERPINA1, MFI2,* and *FOXM1* directly in the PPI network, indicating that *GPC3* might also act in NSCLC by interacting with *SERPINA1, MFI2,* and *FOXM1*. Germline mutation of *MFI2* is significantly greater in LUAD patients among young non-smokers, which may be implicated in the pathogenesis of LUAD [46]. *FOXM1* functions in cell cycle progression, and increased *FOXM1* expression is related to unfavourable outcomes of NSCLC due to the promotion of cell metastasis [47]. *FOXM1* plays a role in EMT induced by TGF-beta1, and miR-134 functions as an EMT suppressor by targeting *FOXM1* in NSCLC cells [48]. *FOXM1* overexpression contributes to cell invasion and migration in NSCLC, which are responsible for the adverse survival of patients with this disease [49, 50]. *FOXM1* can affect gefitinib resistance in NSCLC cells in vitro, making this gene a target for reducing resistance to gefitinib [51]. These results suggest that *FOXM1* may be correlated with the prognosis of patients with NSCLC. Combined with Cox regression analysis, a risk-score model consisting of the prognosis-associated genes *MFI2*, *FOXM1*, and *GPC3* was developed for LUAD prognosis, and the AUC values of the 1-year, 3-year, and 5-year survival situations were stabilised at approximately 0.6, suggesting a relatively higher reliability.

In LUSC, the expression of *GPC3* was not significantly related to survival time (*P* > 0.05). Therefore, a risk-score model consisting of the prognosis-associated genes *SERPINA1* and *FOXM1* was established for LUSC. α-1 antitrypsin (AAT) is a serine proteinase inhibitor that plays an antiprotease protective role in the human body, and mutations in the gene *SERPINA1* can lead to chronic obstructive pulmonary diseases by inducing AAT deficiency [52]. The *SERPINA1* PiMZ genotype, combined with smoking, causes lung-function decline by modifying the correlation between longitudinal change and occupational exposure in lung function [53]. Increased *SERPINA1* gene expression ameliorates tumour cell migration, apoptosis resistance, and colony formation, and *SERPINA1* and its corresponding protein, AAT, influence the mechanisms of lung cancer [54]. Thus, *SERPINA1* may influence the outcomes of NSCLC patients.

## Conclusion

In conclusion, *GPC3* was significantly downregulated in NSCLC tissues compared with that in paracancer tissues, and its expression in LUAD was significantly lower than that in LUSC. In LUAD samples, *GPC3* expression was significantly correlated with pathologic N, pathologic T, gender, and tumour stage. The PPI network showed that GPC3 can interact with SERPINA1, MFI2, and FOXM1 directly. In addition, the three-gene risk-score model (involving *MFI2*, *FOXM1*, and *GPC3*) for LUAD and the two-gene risk-score model (involving *SERPINA1* and *FOXM1*) for LUSC might be useful in predicting the prognosis of tumour patients. However, the utility values of the risk-score models should be further validated in subsequent experiments.

## Data Availability

The data used for analysis in this study are all from the University of California Santa Cruz (UCSC) Xena database (https://xenabrowser.net/datapages/).

## Abbreviations

GPC3: Glypican 3
NSCLC: Non-small-cell lung carcinoma
LUAD: Lung adenocarcinoma
LUSC: Lung squamous cell carcinoma
DEGs: Differentially expressed genes
SCLC: Small-cell lung carcinoma
LCLC: Lung large cell carcinoma
*AEG-1*: Astrocyte-elevated gene-1
*MMP9*: Matrix metalloproteinase-9
*GAPDH*: Glyceraldehyde-3-phosphate dehydrogenase
*HIF-2α*: Hypoxia-inducible factor-2α
*NDRG2*: N-MYC downstream-regulated gene 2
*Notch2*: Notch homolog 2
*CLDN3*: Claudin-3
EMT: Epithelial–mesenchymal transition
HCC: Hepatocellular carcinoma
PPI: Protein–protein interaction
GO: Gene ontology
KEGG: Kyoto encyclopedia of genes and genomes
OS: Overall survival
KM: Kaplan–Meier
ROC: Receiver operating characteristic
SERPINA1: Serpin family A member 1
MFI2: Melanin transferrin
FOXM1: Forkhead box M1
