# Correction to: GPC3 affects the prognosis of lung adenocarcinoma and lung squamous cell carcinoma

**DOI:** 10.1186/s12890-021-01715-z

**Published:** 2021-11-22

**Authors:** Jing Ning, Shenyi Jiang, Xiaoxi Li, Yang Wang, Xuhong Deng, Zhiqiang Zhang, Lijie He, Daqing Wang, Youhong Jiang

**Affiliations:** 1 Molecular Oncology Department of Cancer Research Institution, The First Hospital of China Medical University, Nanjingbei Street, Heping District, Shenyang, 110001 Liaoning Province China; 2 Department of General Medicine (VIP Ward) and Department of Tumor Supportive and Palliative Medicine, Cancer Hospital of China Medical University, Liaoning Cancer Hospital and Institute, No.44 Xiaoheyan Road, Dadong District, Shenyang, 110042 Liaoning Province China; 3 Department of General Practice, The First Hospital of China Medical University, Nanjingbei Street, Heping District, Shenyang, 110001 Liaoning Province China; 4 Central Laboratory, Cancer Hospital of China Medical University, Liaoning Cancer Hospital and Institute, No. 44 Xiaoheyan Road, Dadong District, Shenyang, 110042 Liaoning Province China; 5 The People’s Hospital of Liaoning Province, No.33 Wenyi Road, Shenhe District, Shenyang, 110016 Liaoning Province China

## Correction to: BMC Pulm Med (2021) 21:199 https://doi.org/10.1186/s12890-021-01549-9

Following publication of the original article [1], it was brought to the authors’ attention that the control group and the LUSC group in Fig. 8 had been mislabelled and that the descriptions of A and B in the figure legend in Fig. 9 had been reversed, with the result that there were errors in the article content associated with these figures.

The figures and the related content have now been corrected in the published article, and the corrected figures and corrected content can be found detailed below:

**Fig. 8:**
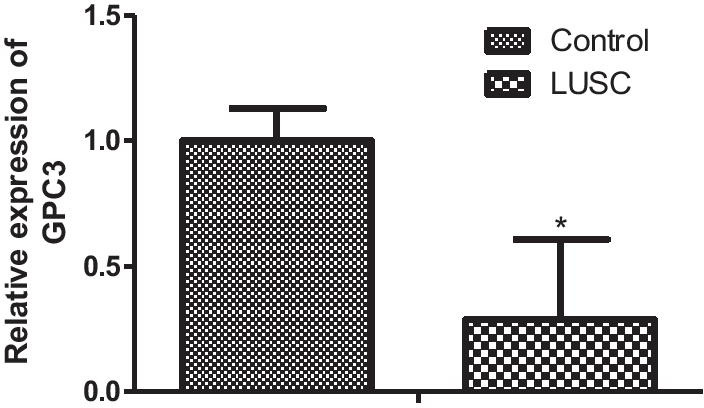
Relative mRNA expression by quantitative real-time PCR of GPC3 in LUSC compared with control group, *P < 0.05

**Fig. 9:**
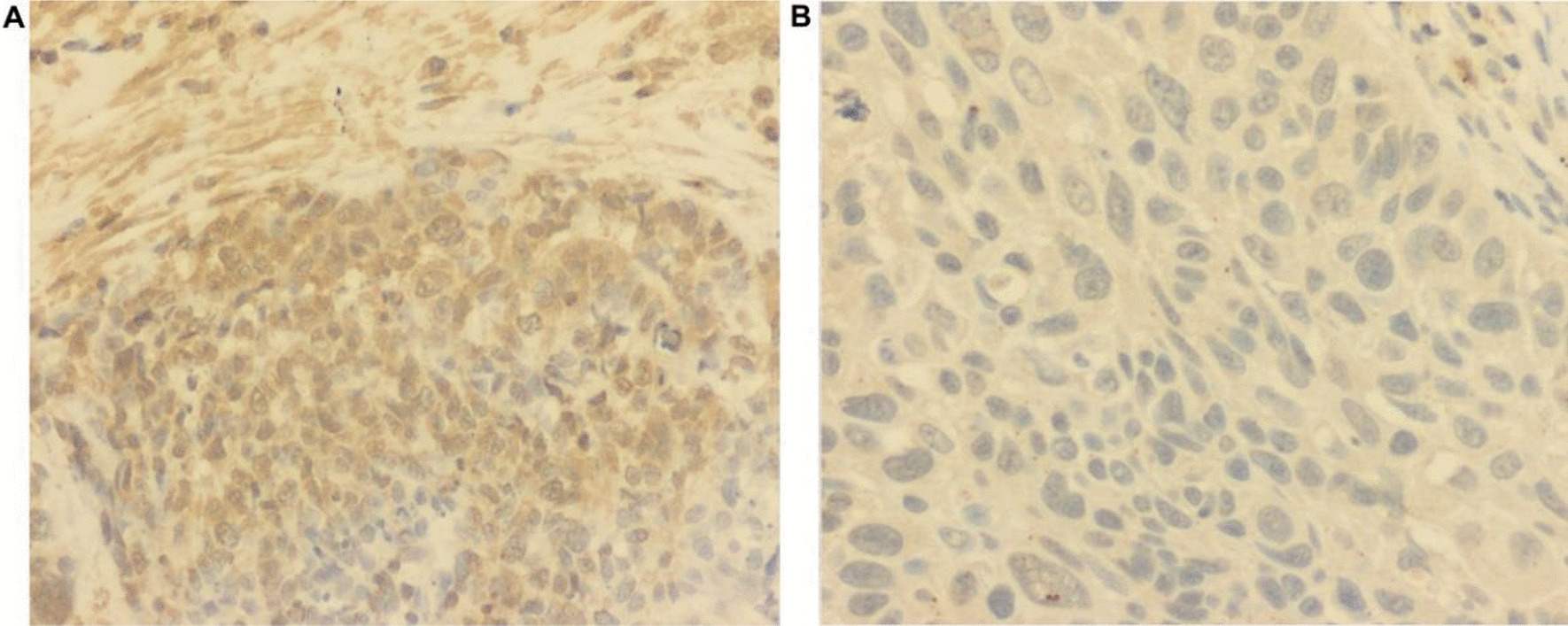
Representative photos of positive immunohistochemical *GPC3* expression in LUSC tissues (**A**) and negative *GPC3* expression in LUSC tissues

**Abstract**

“*Results:* The qRT-PCR result showed that GPC3 expression was much lower in the LUSC tissues than that in the control group. IHC results further showed that GPC3 was more negatively expressed than positively expressed in LUSC tissues.”

**Methods**

“The paraffin sections of cancer tissues were collected from 10 patients undergoing pulmonary malignant tumour surgery at the Liaoning Cancer Hospital and Institute between June 2018 and June 2020.”

**Results**

“*GPC3* expression was much lower in the LUSC tissues than that in the control group (*P* < 0.001, Fig. 8).”

“IHC analysis revealed negative staining of *GPC3* protein in seven of the ten (70%) paraffin-embedded LUSC tissues, while positive staining was observed in the remaining cases (three of ten, 30%) (Fig. 9). This suggests that the low expression of *GPC3* may be related to the occurrence of LUSC.”

The authors thank you for reading this correction and apologize for any inconvenience caused.
